# Significant non-existence of sequences in genomes and proteomes

**DOI:** 10.1093/nar/gkab139

**Published:** 2021-03-10

**Authors:** Grigorios Koulouras, Martin C Frith

**Affiliations:** Artificial Intelligence Research Center, National Institute of Advanced Industrial Science and Technology (AIST), 2-3-26 Aomi, Koto-ku, Tokyo 135-0064, Japan; Artificial Intelligence Research Center, National Institute of Advanced Industrial Science and Technology (AIST), 2-3-26 Aomi, Koto-ku, Tokyo 135-0064, Japan; Graduate School of Frontier Sciences, University of Tokyo, Kashiwa, Chiba, Japan; Computational Bio Big-Data Open Innovation Laboratory (CBBD-OIL), AIST, Shinjuku-ku, Tokyo, Japan

## Abstract

Minimal absent words (MAWs) are minimal-length oligomers absent from a genome or proteome. Although some artificially synthesized MAWs have deleterious effects, there is still a lack of a strategy for the classification of non-occurring sequences as potentially malicious or benign. In this work, by using Markovian models with multiple-testing correction, we reveal significant absent oligomers, which are statistically expected to exist. This suggests that their absence is due to negative selection. We survey genomes and proteomes covering the diversity of life and find thousands of significant absent sequences. Common significant MAWs are often mono- or dinucleotide tracts, or palindromic. Significant viral MAWs are often restriction sites and may indicate unknown restriction motifs. Surprisingly, significant mammal genome MAWs are often present, but rare, in other mammals, suggesting that they are suppressed but not completely forbidden. Significant human MAWs are frequently present in prokaryotes, suggesting immune function, but rarely present in human viruses, indicating viral mimicry of the host. More than one-fourth of human proteins are one substitution away from containing a significant MAW, with the majority of replacements being predicted harmful. We provide a web-based, interactive database of significant MAWs across genomes and proteomes.

## INTRODUCTION

The terms *minimal absent words* (MAWs), *nullomers* and *primes* all describe sequences that do not occur in the entire genome or proteome of an organism. Primes are the shortest sequences that are not found across all known species, whereas nullomers are the shortest possible absent motifs in a species (1,2). The broader term MAW includes both nullomers and longer absent sequences which share a common characteristic: becoming present after removing either their leftmost or rightmost letter (2). Although many biotechnological applications have been envisioned, from potential selective drugs (3,4) to forensic practice (5), the actual role of MAWs has intensely been debated (6–8) and still remains enigmatic. Lately, fast tools and efficient algorithms have been introduced making the discovery of globally missing sequences practical (9–15). In 2012, Alileche and colleagues demonstrated that two absent 5-amino-acid peptides cause fatal damage to cancer cells (4), while 5 years later, Alileche and Hampikian showed that the same MAWs have a broad lethal effect on cancer cell lines derived from nine organs (3). In a recent study on over 30 species, Georgakopoulos-Soares *et al.* reported more nullomers than expected by chance, suggesting negative selection against them (16), whereas in 2012, Patel and colleagues reported that rare or non-existent oligopeptides can enhance immune response. They additionally showed that exogenously added non-occurring 5-mers in adjuvant vaccines positively contribute to antigen-specific immune activation (17). Silva and colleagues have reported that three minimal 12-nucleotide fragments entirely absent from the human genome, appear consistently at the same location in two protein-coding genes of Ebola virus genomes (18). In the same study, the term minimal *relative absent words* (RAWs) is introduced, describing sequences that are present in a pathogenic organism but absent from its host. In the same vein, in a recent study expansion, Pratas and Silva revealed the absence of four human genomic nullomers, which persistently occur in genomic sequences of the SARS-CoV-2 virus and triggered a discussion about the potential utilization of RAWs for rapid diagnostics and novel therapeutics (19). Although there exists an unambiguous sequence conservation in their findings, it is not clear whether the absence of these oligomers is statistically expected. On one hand, all the above findings ideally support the conjecture that MAWs may have gone extinct due to evolutionary pressure or putative deleterious effects. Perhaps the unfavourable properties of MAWs are linked with forbidden spatial conformations followed by functional consequences incompatible with life. Hence, the structural arrangements of globally absent motifs and the putative perturbation of molecules upon their appearance (i.e. emergence of a MAW upon a mutation) form an interesting area for future research. Conversely, a finite set of sequences, for example the entire genome or proteome of a species, does not include all the different combinations of elements from the alphabet it is composed of, due to the fact that the combination of residues in a sequence increases exponentially with its length. Therefore, it still remains a riddle whether the absence of a MAW is, in fact, an evolutionary consequence linked with adverse effects or a product of randomness. In short, there is a range of possible explanations for MAWs: they could be missing purely by chance, or truly forbidden (e.g. lethal to the organism), or they could reflect sequences that are disfavoured but not totally forbidden. Disfavoured sequences would have an increased probability of being absent by chance.

In this study, we introduce a robust probabilistic method named *Nullomers Assessor* (https://github.com/gkoulouras/nullomers-assessor) for the evaluation of absent oligomers in any species, considering the fact that biological sequences of living organisms are driven by mutational biases and natural selection, and consequently are not entirely random (20). Naturally occurring sequences present patterns and combinatoric properties which can be signatures for the identification of functional elements as such promoters, tandem repeat expansions, introns, exons and regulatory elements (21). In addition, evolutionarily well-separated species are known to possess distinct statistical characteristics in their DNA or peptide sequence chains (22). All these distinctive properties of biological sequences have frequently been studied using probabilistic models. Markov chain models have been widely and successfully employed in various biological problems including sequence analysis in the past (23–26). Taking advantage of these properties of biological sequences, we developed a method which approximates the likelihood of an absent sequence to occur exactly zero times, in order to address the following three questions. First, are there statistically significant minimal absent sequences in biological species; in simple words, what is the expected probability for an actual MAW to be indeed absent, based on the compositional pattern in the full genome or proteome of a species? Second, are there significant MAWs in common across evolutionarily diverse living organisms? And finally, does the creation of a previously absent sequence perturb a molecule; more precisely, are there mutations with functional or stability impact which at the same time generate a missing word?

Furthermore, given the possible relevance of MAWs to diverse research areas, as for example the under-studied ‘dark’ majority of the genome (27,28), patterns and evolutionary features between viruses and host species (29–33), rare variants (34–36) as well as cancer driver mutations in non-coding regions (37,38), we provide the community with the results of the present study in the form of a publicly-available, downloadable and web-accessible repository of significant absent motifs, named *Nullomers Database* (https://www.nullomers.org/). Modern web-technologies and visualization features have been harmonically combined resulting in a dynamic and user-friendly environment. To the best of our knowledge, this is the first attempt for a centralised, open-access and searchable resource of non-occurring genomic and peptide sequences. *Nullomers Database* is intent on being a periodically updated and continuously enriched repository of significant absent sequences from various organisms, as they have been assessed by *Nullomers Assessor*. We are hopeful that the intuitive and interactive graphical user interface of *Nullomers Database*, in conjunction with the integrated annotation and the powerful searching features that it employs, will facilitate exploration and shed new light on the puzzling and, up to the present time, little-known world of MAWs.

## MATERIALS AND METHODS

### Identification of minimal absent words

The identification of MAWs was achieved using the MAW console application (9), an open source *O*(*n*)-time and *O*(*n*)-space algorithm for finding minimal absent words based on suffix arrays. When applied to long sequences of size $n$ the algorithm requires more than $20*n\;$bytes of RAM, which causes a bottleneck on large datasets such as the human genome. For the detection of MAWs on sizable datasets we used the em-MAW software tool (10), a marginally slower alternative which utilises external memory. Both MAW and em-MAW require an input fasta file which contains the whole genome or proteome of an organism, as well as two numerical arguments that indicate the shortest and longest MAWs to search for. Throughout the study, we searched for sequences of length between 4 and 14 nucleotides which are absent from both forward and reverse-complement strands. For peptide sequences we set the identification range between minimum 4 and maximum 6 amino acids in order to keep complexity at a reasonable level. The output of both MAW and em-MAW is a list of missing sequences of a given dataset.

In our analysis, we downloaded full proteomes of two main organisms (i.e. *Homo sapiens* and *Mus musculus*) from UniProt (39; https://www.uniprot.org/proteomes/), while a range of >1500 genomes from archaea, bacteria, protozoa, fungi, invertebrates and vertebrates were retrieved via NCBI Genome (40; ftp://ftp.ncbi.nlm.nih.gov/genomes/). We developed custom Python scripts to discard headers and concatenate sequences from multiple fasta files, to produce files with one header and one-line sequence for each organism. The above step was applied to protein sequences as well because MAW and em-MAW are developed in a way to calculate minimal absent words of each individual record in a fasta file. For the identification of peptide MAWs, we combined information both from the Swiss-Prot and TrEMBL sections of UniProtKB including protein isoforms. The incorporation of predicted with manually reviewed records, including variant isoforms, produces a list of more confident non-occurring sequences. The final pre-processing step included the removal of any ambiguous residues (character N in genomes or B, J, X, Z in proteins). Eventually, we generated lists of MAWs from various species which were used as an input for downstream analysis in *Nullomers Assessor*.

### Applying Markov models to genomic and protein sequences

Statistical models represent the observed variability in data by probability distributions. A simple model of a sequence $X\; = \;( {X1,\;X2,\;X3,\; \ldots } )\;$is a first-order Markov chain, where each position is dependent only on its immediate precursor. For example, the probability of observing a ‘G’ at one position depends (only) on whether there is a ‘C’ in the previous position. This can be expressed as:

(1) $$\begin{eqnarray*}P({Xn|X1, \ldots ,Xn - 1}) = P({Xn|Xn - 1}),{\rm{\;for\;all\;}}n \ge 2 \end{eqnarray*}$$

In our method, we consider genomes and proteomes (hereinafter background sequence) as Markov chains. A first order Markov chain is a model where each position is contingent merely on its previous position. Likewise, in an $n$th order Markov chain, each position depends on the $n$ previous positions. In order to decide whether a MAW is statistically expected to exist, we estimate Markov probabilities. First, the frequencies of elements (nucleotides or amino acids) of the background sequence are calculated. Then, three Markov probability matrices are generated, one for each of the first three Markov model orders. In general, a substitution matrix for $m$ distinct letters (e.g. $m\; = \;4$ for DNA) and order $n$, is a grid of $m\times{m^n}$ probabilities. As an example, a stochastic matrix of third-order for all the naturally occurring amino acid residues in a bacterium (20 distinct amino acids assuming that neither selenocysteine nor pyrrolysine are present) requires a matrix of $20\times{20^3} = \;160\,000$ cells (namely $20\;$rows and ${20^3}$ columns, or vice versa). Each of these $160\,000\;$probabilities indicate the likelihood for a specific amino acid to occur given the three preceding amino acids.

In non-mathematical terms, each residue of a biological sequence is dependent on the $n$ previous elements, where$\;n$ defines the order of a Markovian process. Implicitly, a stochastic process in a biological sequence can reveal the sequential preferences among neighboring residues as well as reflect avoided motifs which may introduce an unfavorable structural folding. We utilised the above-described fundamental mathematical notion and developed a custom Python script (*Nullomers Assessor*) which approximates the likelihood for a MAW to occur zero times based on the first four orders of Markovian chains (including zeroth order). More precisely, four distinct *P*-values are assigned to each MAW of the list. To elucidate and put this probabilistic property into a more biological context, we provide the following example. Assuming the peptide ‘PTILA’ is an absent minimal 5-mer, then the probability of being entirely absent based on a second-order Markov chain can be calculated as illustrated below:

(2) $$\begin{equation*}P\;({{\rm one}\;{\rm occurrence}})= P(P)*P({T|P})*P({I|PT})*P({L|TI})*P({A|IL})\end{equation*}$$

(3) $$\begin{eqnarray*}{\rm expected}\;{\rm number}\;{\rm of}\;{\rm occurrences} &=& P({{\rm one}\;{\rm occurence}})\nonumber\\ &&*\;({{\rm sequence}\;{\rm length} - {\rm nullomer}\;{\rm length} + 1}) \end{eqnarray*}$$

(4) $$\begin{equation*}P\left( {{\rm zero}\;{\rm occurences}} \right) \approx {\rm{exp}}\left( { - \;{\rm expected}\;{\rm number}\;{\rm of}\;{\rm occurrences}} \right)\end{equation*}$$

Initially, the probability of ‘PTILA’ to occur is estimated (Equation 2). In this formula, *P*(*P*) denotes the observed frequency of proline in the entire background sequence. Subsequently, *P*(*T*|*P*) signifies the probability of threonine to arise after a proline. Similarly, *P*(*I*|*PT*) indicates the probability of isoleucine to occur given the previous two adjacent amino acids are a proline followed by a threonine, and so forth. Next, the expected number of occurrences for the specific actual MAW is estimated (Equation 3), a value that is used for the final estimation of the second order zero-occurrence probability (Equation 4). In a similar manner, the probabilities of first and third orders are computed, while the zeroth order simply mirrors the frequency of residues in the background sequence. In simple words, the calculated *P*-values represent the probability of a minimal absent word to be indeed absent based on the rate at which the residues occur as well as three additional transition probabilities which reflect the frequency of 2-, 3- and 4-mers of the examined genome or proteome.

Although four different probabilities are calculated, the maximum *P*-value is kept and assigned to each examined MAW. Since a *P*-value denotes the chance of a MAW to occur exactly zero times (namely to be absent), then the lower the value, the more expected the MAW is to exist (or equivalently the less expected it is to be, indeed, absent). By keeping the highest probability amongst the four calculated *P*-values, we expect to end up with fewer but more confidently true-positive results.

### Multiple hypothesis testing and statistical correction

There is a large number of short sequences that each have a chance of being absent. Therefore, the emergence of false positive results must be controlled (41). More specifically, all the four calculated *P*-values of each MAW are corrected for Type I errors and readjusted based on one of three statistical correction methods, which are provided built-in with the current version of the tool. In exact terms, users can choose between Bonferroni (42), Benjamini–Hochberg (43; widely known as false discovery rate or simply FDR), or Tarone method (44) which is a modified Bonferroni procedure. The Bonferroni correction is particularly conservative, especially when applied to proteome datasets, due to the large alphabet size and high number of tests. More specifically, each of the four individual *P*-values is multiplied by the count of all possible different $k$-mers of length $k$. For example, the corrected *P*-value of a genomic $k$-mer is the product of the actual *P*-value multiplied by ${4^k}$, where $k\;$denotes the length of the examined absent motif. For peptide MAWs the multiplier changes to ${20^k}$. Generally, the following formula illustrates the Bonferroni correction step:

(5) $$\begin{equation*}q{\rm value}\; = \;p{\rm value}*\;{{\rm {\rm alphabet}}^{{\rm length}}}\end{equation*}$$

Alternatively, in the Benjamini–Hochberg procedure, the probabilities are sorted in descending order and sequentially rejected if the product of a *P*-value and the number of remaining tests is greater than a cut-off limit. The FDR method though, which constitutes a milder alternative, performs markedly loosely when applied to large eukaryotic genomes resulting in thousands of significant MAWs. This motivated us to incorporate a third correction option, the Tarone methodology. This is a special case of the Bonferroni method, where we make it less conservative by doing fewer tests. For a given word-length $k$, we calculate the zero-occurrence probability (the maximum of the four Markov probabilities) for each of the ${4^k}\;$$k$-mers. Then, these ${4^k}$$k$-mers are ordered in descending order of zero-occurrence probability. Next, we exclude from testing any $k$-mer whose absence would not be significant because the Bonferroni-adjusted *P*-value is above a cut-off threshold:

(6) $$\begin{equation*}q{\rm value}\; = \;p{\rm value}*\;({{\rm alphabet}^{{\rm length}}} - \;{\rm counter})\end{equation*}$$

Equation (6) represents the mathematical notation of the Tarone method, where *counter* denotes a number which progressively increases by 1 when a $k$-mer is excluded from testing. Finally, the ‘testable’ $k$-mers that remain are compared to the actual MAWs in the list, and those in the intersection are output. In this way, the stringent nature of Bonferroni remains, whilst a milder adjustment is performed every time a test is excluded due to impossibility of significance.

Next, we tested the three different correction methods using a common dataset and a constant threshold of false discovery control. The results show that Bonferroni correction performs the strictest cleansing of false positive MAWs (very likely at the cost of increased false negatives), followed by the Tarone method, whereas the FDR approach constitutes the least stringent alternative. More precisely, any result-set derived by Bonferroni is a subset of the corresponding Tarone set, while the results of the FDR method almost always include all the above outcomes. Throughout our analysis, a fixed false discovery threshold of 1% has been applied, both when searching for genomic or peptide MAWs. In order to eliminate the emergence of Type I errors to the utmost degree, we report a MAW as significant only when all four corrected probabilities are lower than the user specified cut-off.

### Testing precision by shuffling input sequences

Random sequence shuffling is a widely used approach to evaluate stochasticity as well as statistical significance of results. In order to evaluate the rigour of our method, especially because Equation (4) is only approximately true, we performed permutation tests by randomly shuffling the human proteome. Since *Nullomers Assessor* calculates up to third-order probabilities, we sought to retain unaltered not only the counts of distinct amino acids, but also higher-order statistics, such as the frequency of adjacent letters (doublets and triplets) of the entire proteome. For this purpose, we used a sophisticated shuffling algorithm, uShuffle (45), which performs random shuffling of sequences while preserving $k$-let counts. The C# software package of the uShuffle method was used to shuffle the human proteome 10 times while preserving the frequency of amino acids, doublet occurrences and tripeptides. Next, for each of the 10 shuffled proteomes we re-generated lists of MAWs in order to examine whether any of the new random absent sequences would come into view as significant. By keeping the singlet, doublet, and triplet amino acid frequencies unchanged but not their order, we expected to end up with utterly different lists of MAWs. We used the original human proteome as a background sequence and re-ran our method 10 times (once per each new list of MAWs) in order to assess the newly created lists of ‘counterfeit’ absent words. Even though the script was executed using identical parameters (background proteome, correction method, threshold of statistical significance), no significant results emerged in any of the 10 attempts, demonstrating the stringency of our methodology. Thus, we assessed 10 sets of random missing sequences using the real transition probabilities of the reference human proteome given the fact our method scans any background sequence by considering frequencies up to 4-mers (precisely up to third-order Markov chains). The outcome of this step suggests that *Nullomers Assessor* is able to disclose truly significant MAWs.

## RESULTS AND DISCUSSION

In the present study, we rigorously assess absent sequences for their statistical significance. We examine genomes and proteomes from hundreds of organisms (Table 1) and show lists of MAWs which are unexpected to be absent, in contrast with other missing sequences. Our findings demonstrate that several thousands of absent sequences are statistically expected to occur in various genomes. The longest significant human genomic MAW is composed of 13 nucleotides, whilst all the significant peptide MAWs from the same organism are five residues long. After applying our method to the entire human genome, 13 significant genomic MAWs stood out (Bonferroni correction at 1% cut-off) from a set of >27 million non-occurring oligomers. In essence, the specific 13 missing words are highly statistically foreseen to occur somewhere in the human genome but, in reality, they are totally absent. In a similar manner, we analysed peptide MAWs from the human and mouse proteomes. Thirteen absent peptides from the human proteome were classified as significant when the Tarone correction was used, while eight peptides emerged when we applied identical parameters to the mouse proteome (Table 3). Moving the hypothesis of harmfulness a step forward, we systematically explored MAW-making mutations which are one residue away from the reference sequence. More specifically, we calculated all the possible single amino acid substitutions in all protein records of each proteome that can give rise to any of the total 21 significant absent words. This might offer useful insights for unravelling plausible mechanisms of evolvability that underlie peptide MAWs. Prior research suggests that different residues differ in respect of their mutational preference (46) and reports implications in phosphorylation sites (47). Therefore, the mutational landscape of MAWs presented in this work, may provide a useful resource for future sequencing studies, especially in the field of proteomics (48). To this end, we highlight and share more than 30.000 candidate MAW-making alterations in the form of interactive visual components via *Nullomers Database* web-portal. We compute and display pathogenicity predictions for all MAW-making mutations and extract a list of probably damaging and, simultaneously, disease-implicated mutations (Supplementary Spreadsheet 1). In addition, we make available a complementary catalogue of 176 curated phosphorylation sites (Supplementary Spreadsheet 2) which can lose their phosphorylation ability upon a mutation and, simultaneously, generate a MAW. Next, we show that the most frequent significant absent words in viral sequences are restriction recognition sites indicating that viruses have probably got rid of these motifs to facilitate invasion of bacterial hosts. It is worth noting that not every species or virus has significant MAWs, thus the provided result-set can be used to reduce the vast space of absent motifs and prioritise non-occurring sequences for future research questions. Finally, we share lists of human MAWs which are seldomly present in viruses suggesting molecular mimicry between virus and host (Supplementary Spreadsheet 3).

**Table 1.** Summary table outlines the number of analysed genomes and the count of identified significant MAWs per division

Division	Number of species	Number of total significant MAWs	Number of unique significant MAWs
Archaea	144	2419	1074
Bacteria	559	20 082	10 547
Fungi	14	160	159
Invertebrate	19	857	711
Plant	52	4047	3980
Protozoa	13	104	102
Vertebrate mammalian	43	471	451
Vertebrate other	56	3032	2416
	900	31 172	19 440

### Genomic MAWs across evolution

**Figure 1.**
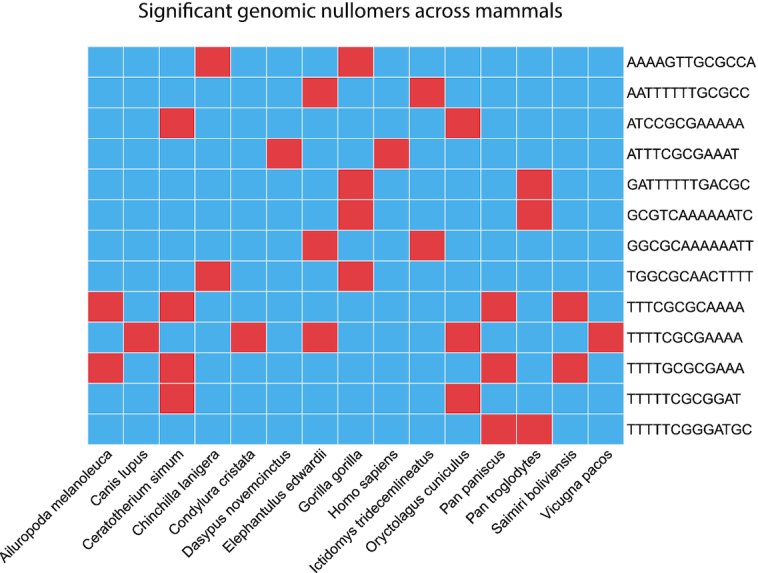
Comparison of significant genomic absent sequences across mammals. Only MAWs that are shared in at least two species are shown. A red grid-cell indicates a significant MAW (evaluated by *Nullomers Assessor*) while blue colour denotes a non-significant or present motif.

**Figure 2.**
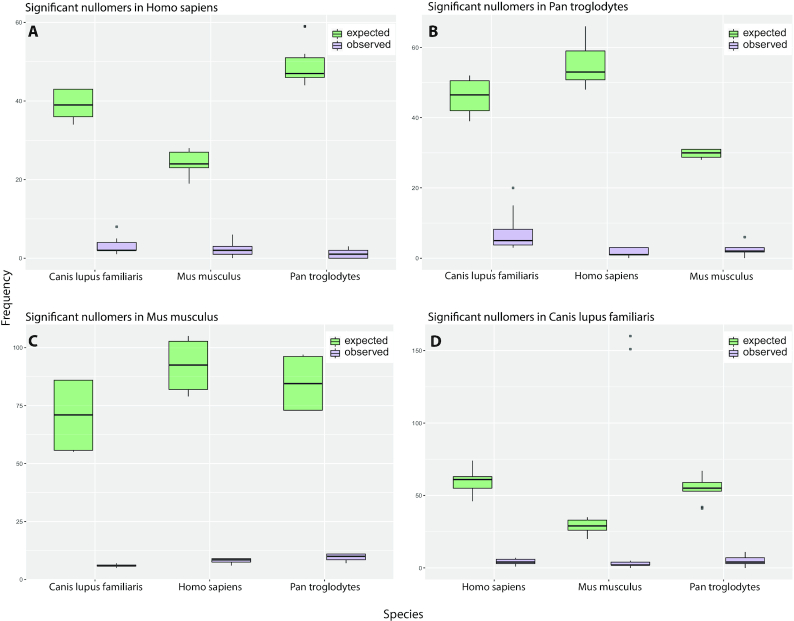
(**A**) Frequencies of 13 human genomic MAWs in *Pan troglodytes*, *Mus musculus* and *Canis lupusfamiliaris*. The green boxplots show the expected count of each human MAW in each non-human species, while the purple boxes correspond to the observed frequencies. Similarly in (**B**) the MAWs of chimpanzee have been searched against the genomes of *Homo sapiens*, *M. musculus* and *C. lupusfamiliaris*. In (**C**) and (**D**), the MAWs of *M. musculus* and *C. lupusfamiliaris*, respectively, have been searched against the other 3 species.

To assess whether MAWs have an ancestral origin, we examined a plethora of organisms ranging from bacteria to human. We hypothesized that distantly-related genomes would share fewer similar sequence features and therefore one would not be surprised to find fewer or no MAWs in common. In contrast, despite the stringent filtering criteria of our methodology, we would expect to end up with identical significant MAWs from closely-related organisms due to the existing high similarity both in genomic and protein sequences. Surprisingly, while some significant motifs are sporadically shared by some mammals, most are not shared by closely-related mammals (Figure 1). Although none of the 13 genomic MAWs in *Homo sapiens* have emerged significant in *Pan troglodytes*, despite their genomes being ∼98% identical, the latter shares three absent words with the closely related species *Gorilla gorilla* and *Pan paniscus*. Furthermore, *Pan paniscus* shares two MAWs with *Saimiri boliviensis* forming a cluster of related organisms with significant results in common. This led us to investigate whether significant absent sequences in human are present in chimp, and vice versa. Since the two genomes are very similar, it might be considered not surprising that absent words in human are rare in chimp. For this reason, we also investigated two more distant species, *Mus musculus* and *Canis lupus familiaris*. Then, for each significant MAW of a species, we computed the observed as well as the expected number of occurrences in the other three organisms by exploiting again equations (2) and (3) in the *Materials and Methods* section. We found that most *Homo sapiens* MAWs are present in *Pan troglodytes* (and vice versa) with a median frequency of one occurrence, while the median number of expected occurrences of the significant human MAWs in chimp is 47 (Figure 2A). In a like manner, the estimated median frequency of chimp-derived MAWs in the human genome is 53 (Figure 2B) while the detailed dataset is provided in Supplementary Spreadsheet 4. Figure 2C and D outlines a similar trend in MAWs of *M. musculus* and *C. lupus familiaris*, respectively, where the expected number of occurrences is again more than the actual observations. This finding suggests an alternative hypothesis in which significant MAWs are not completely forbidden, but they are strongly suppressed. Strongly suppressed sequences are expected to occur just a few times, so by chance fluctuations some of them could appear zero times.

**Figure 3.**
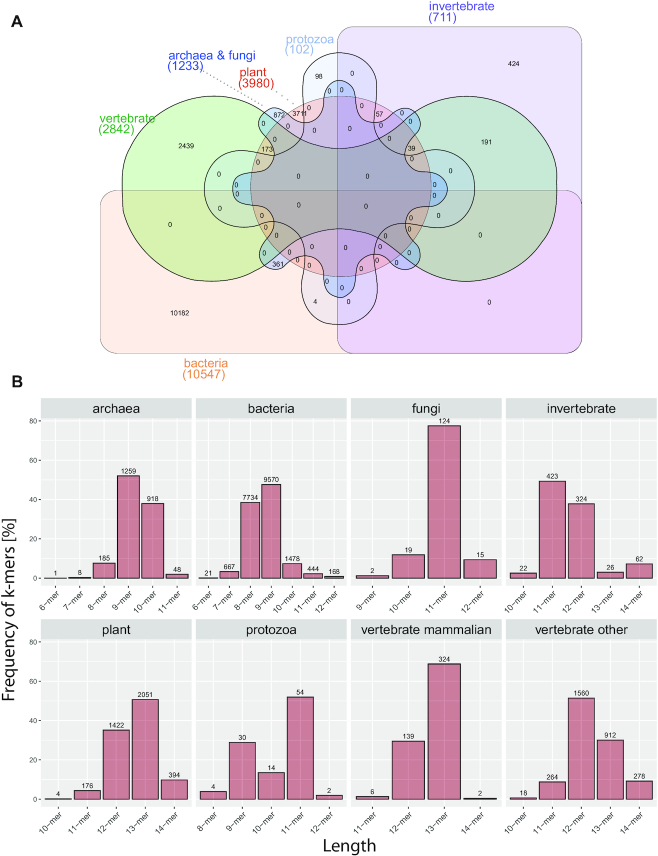
(**A**) Venn diagram showing the number of shared MAWs derived from 900 genomes grouped by division. Created using InteractiVenn (89; http://www.interactivenn.net/index2.html). (**B**) Distribution of MAW length per division. Each bar represents the count of MAWs for a specific motif length.

In contrast, none of the ∼2850 unique significant MAWs in vertebrates are shared with any of the other species, which belong to archaea, bacteria, protozoa, or fungi (Figure 3A). To some extent, this may be due to the heterogenous complexity among species of different kingdoms because significant MAWs of vertebrates are usually longer (Figure 3B). Overall, these observations are in accordance with findings reported by Acquisti *et al.* (6). The authors of that study have demonstrated that species with a more-recent common ancestor share more MAWs in common in comparison with more distantly-related organisms. Here, we provide evidence that more-closely related organisms share not only common deficits in general, but also some identical significant missing patterns, supporting the hypothesis of evolutionarily-conserved aversion to these sequences.

Furthermore, we observed multiple genomic MAWs in common across species ranging from 6 to 14 bases. Table 2 presents the most frequent significant nucleotide sequences whose absence is shared between at least 18 species, while a complete list is in Supplementary Spreadsheet 5. Thus, identical significant MAWs which are shared across species might be usable for biotechnological applications or further research. What is noticeable in this dataset is the high rate of mononucleotide tracts. It has previously been shown that enrichment of poly(A) tracts is linked with important functional roles, including DNA methylation (49), ribosome stalling (50), and translational efficiency (51), to name a few. The longest poly(A) and poly(T) sequences in our dataset are an 11-nucleotide motif absent in 18 bacterial and archaeal species, while a 12-mer poly(T) tract has been marked significant in *Clostridium sartagoforme*, implying the high content of repeated adenine and thymine in their genomes. In *H. sapiens*, the ‘ATTTCGCGAAAT’ MAW constitutes one of the nearly 250 palindrome MAWs of the result set and it is shared with *Dasypus novemcinctus* and *Gekko japonicus*. The frequency of mononucleotide tracts in significant MAWs is shown in Supplementary Material: Table 1, while all the palindromic MAWs can be easily accessed and downloaded via *Nullomers Database*.

**Table 2.** The most frequent significant MAWs across species (this includes reverse-complement MAW pairs, whose significance estimates may differ slightly, based on sequence composition of one DNA strand)

MAWs	Length	Number of species	Division
**GCGCGCGCGCGC**	12	36	Bacteria
**CGCGCGCGCGCG**	12	35	Bacteria
**AGGCGCCT**	8	31	Archaea & bacteria
**TTTTTTTTTT**	10	22	Archaea & bacteria
**GGGCCCCCCC**	10	21	Bacteria
**GGGGGGGCCC**	10	21	Bacteria
**AAAAAAAAAA**	10	19	Archaea & bacteria
**TAATTCGAA**	9	19	Bacteria
**TTCGAATTA**	9	19	Bacteria
**AAAAAAAAAAA**	11	18	Archaea & bacteria
**AGAGGCGCC**	9	18	Archaea & bacteria
**GCCCCCCCC**	9	18	Archaea & bacteria

### Peptide MAWs and MAW-making mutations

Since a MAW is a minimal-length absent sequence, a proteome will have sites that can generate a MAW upon a single amino acid replacement. To illustrate this point, we provide the following example. *BRCA1* is a human gene that produces a tumour suppressor protein of 1863 amino acids (UniProt ID: P38398) involved in various biological mechanisms including DNA damage repair and embryonic development (52,53). Two possible MAW-making mutations Ser > Pro and Lys > Asn in positions 628 and 1171 can give rise to the ‘LVVPR’ and ‘INESS’ absent words, respectively. Therefore, the actual amino acid chains ‘LVVSR’ and ‘IKESS’ that normally exist in the reference protein sequence could be considered ‘sensitive’ to putative S628P and K1171N mutations in the fourth and second position, respectively. Moving this simple idea forward in conjunction with the scenario of noxiousness behind entirely missing sequences, we hypothesised that MAW-making mutations would potentially introduce unfavourable effects to these molecules. To this direction, we developed an automated procedure that detects positions in proteins which are susceptible to generate one of the significant identified MAWs upon a single amino acid alteration. We applied this to the entire *Homo sapiens* and *Mus musculus* proteomes ending up with a list of 34 053 positions which are prone to create one of the 21 significant MAWs in both organisms (Table 3D, E). In the human proteome, 21 668 potential alterations can lead to one of the 13 significant minimal absent peptides in 16 045 UniProt IDs (of which 6576 belong to unique manually reviewed records). This suggests that more than one fourth of the human proteome is susceptible to introducing an utterly absent sequence, with a single amino acid alteration. With this information in hand, we investigated evolutionary tendencies of mutability (amino acids to be mutated) and targetability (resulting amino acids upon a mutation) in MAW-making positions (Supplementary Material: Supplementary Figures S1 and S2). A clear propensity is apparent in targeted valine in the human proteome, while the extremely high number of mutations to isoleucine in both organisms constitutes an intriguing observation considering that Ile is one of the rare amino acids (frequency < 5%). At the opposite extreme, five amino acids (cysteine, histidine, methionine, tryptophan, tyrosine) are null targets of human MAW-making mutations, simply because none of the 13 significant MAWs have these amino acids. Similarly, asparagine, cysteine, histidine, methionine, phenylalanine, tryptophan and tyrosine are not MAW-making targets in *Mus musculus*. We produced matrices of mutational transitions in order to further detect possible tendencies (Supplementary Material: Supplementary Figure S2). The resulting plots demonstrate that Leu > Val and Leu > Ile are two prevalent alterations in human, whereas Ala > Ile and Glu > Ile are the most common MAW-creating substitutions in the mouse proteome. Curiously, Leu, Ile and Val are precisely the three branched-chain amino acids. Next, we investigated whether the inclusion of non-reviewed proteins (records from TrEMBL) affects the MAWs' mutational space. What one can see in the same figure is that dominant mutational trends remain unaffected either with or without predicted records. Given that amino acid substitutions are not equally probable due to the genetic code, we also calculated the minimum number of nucleotide substitutions necessary to cause any of the amino acid replacements (Supplementary Material: Supplementary Figure S3). The fact that Leu can be mutated to either Val or Ile merely by one nucleotide substitution, suggests a stronger aversion towards the specific MAW-making replacements compared to a hypothetical Cys > Met alteration which requires an entire codon change.

**Table 3.** List of significant genomic MAWs in (A) *Homo sapiens*, (B) *Mus musculus* and (C) *Pan Troglodytes*. List of significant peptide MAWs in (D) *H. sapiens* and (E) *M. musculus*

	MAW	*q*-value	Correction method
**A**	**Genomic MAWs (*Homo sapiens*)**		
	**TATTATGCGCG**	1.61e-05	Bonferroni
	**TTTCGCGAAATT**	1.64e-05	Bonferroni
	**AATTTCGCGAAA**	2.04e-05	Bonferroni
	**CGCGCATAATA**	2.12e-05	Bonferroni
	**AAATTGGCGCAGG**	8.30e-04	Bonferroni
	**CCTGCGCCAATTT**	8.94e-04	Bonferroni
	**GGCGATTTTTGGG**	9.98e-04	Bonferroni
	**TTTGGGCGCAACA**	1.12e-03	Bonferroni
	**TGTTGCGCCCAAA**	1.71e-03	Bonferroni
	**ATTTTTTACGGGC**	1.96e-03	Bonferroni
	**ATTTCGCGAAAT**	2.39e-03	Bonferroni
	**CCCAAAAATCGCC**	2.92e-03	Bonferroni
	**GCCCGTAAAAAAT**	4.36e-03	Bonferroni
**B**	**Genomic MAWs (*Mus musculus*)**		
	**AGTTTTTTCGGAA**	3.62e-06	Bonferroni
	**TTCCGAAAAAACT**	4.17e-06	Bonferroni
	**AATTTTTTGGGCG**	3.82e-03	Bonferroni
	**CGCCCAAAAAATT**	4.30e-03	Bonferroni
**C**	**Genomic MAWs (*Pan troglodytes*)**		
	**AATTTTTGGCGCC**	3.90e-06	Bonferroni
	**GGCGCCAAAAATT**	4.60e-06	Bonferroni
	**TTTTTCGGGATGC**	2.39e-04	Bonferroni
	**GCATCCCGAAAAA**	2.82e-04	Bonferroni
	**CCGGTGAAAGTTT**	3.98e-03	Bonferroni
	**AAACTTTCACCGG**	4.48e-03	Bonferroni
	**GATTTTTTGACGC**	6.72e-03	Bonferroni
	**GCGTCAAAAAATC**	8.33e-03	Bonferroni
**D**	**Peptide MAWs (*Homo sapiens*)**		
	**TVAER**	4.40e-05	Tarone
	**EQAVP**	0.000102	Tarone
	**TVIEL**	0.001373	Tarone
	**AKITL**	0.001424	Tarone
	**ATPAD**	0.001916	Tarone
	**DLKQV**	0.002061	Tarone
	**ALQVI**	0.002852	Tarone
	**VDEAR**	0.004138	Tarone
	**LVVPR**	0.004417	Tarone
	**ELFGV**	0.005102	Tarone
	**PTILA**	0.006082	Tarone
	**NGLGV**	0.006793	Tarone
	**INESS**	0.007286	Tarone
**E**	**Peptide MAWs (*Mus musculus*)**		
	**GVDLK**	4.51e-05	Tarone
	**IGLRS**	0.001781	Tarone
	**PPAEI**	0.002527	Tarone
	**SAAQI**	0.002562	Tarone
	**EPRRG**	0.002964	Tarone
	**ESDLI**	0.003661	Tarone
	**PPTKS**	0.003701	Tarone
	**IALED**	0.005910	Tarone

Recently, massive efforts have been put forth to prioritize the functional importance of phosphorylation sites (54,55) as well as decipher correlation between mutation and phosphorylation in cancer (56). For this reason, we compiled a curated list of susceptible MAW-making phosphosites retrieved from the PhosphoSite Plus database (57). Our analysis revealed 176 phosphorylation sites of high confidence which are prone to give rise to a MAW (Supplementary Spreadsheet 2), while a similar tendency is notable where valine is the most prominent mutational target (Supplementary Material: Supplementary Figure S4). Future research should be devoted to characterizing the impact of MAW-making mutations, especially when a substitution occurs in post translational modification (PTM) sites.

**Figure 4.**
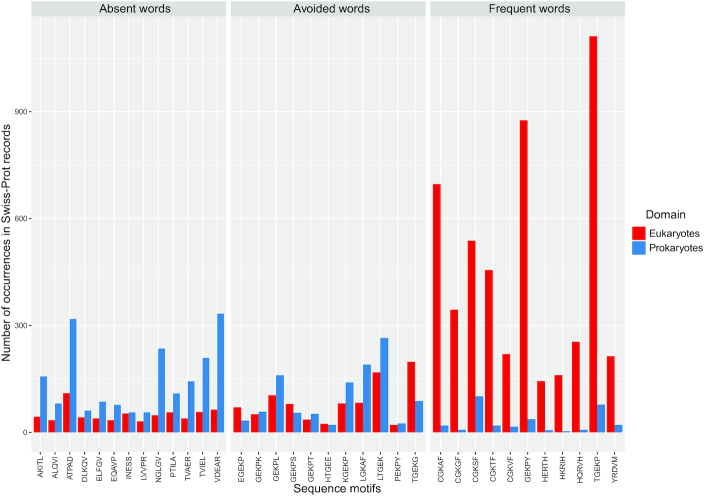
Absent, avoided and frequent pentamers of the human proteome are compared against eukaryotic (metazoa, plant, fungi) and non-eukaryotic (bacteria, archaea, viruses) sequences. The height of each bar indicates the number of occurrences of each motif in eukaryota and prokaryota. The entire Swiss-Prot component of the UniProt database was used as a reference dataset for the analysis.

Rare and non-occurring peptides have been reported to be immunomodulators and vaccine adjuvants (17). A potential explanation of this might be that MAWs are frequent in pathogens but suppressed in the host, hence the immune system is capable of recognising them and responding to threatening pathogens. To this end, we set out to investigate whether significant human peptide MAWs are rare in bacteria, archaea and viruses in contrast to eukaryotic species. Data from the Swiss-Prot component of the UniProt database was used to address this query. For comparison, we performed the same analysis on present-but-avoided and highly-frequent sequences in the human proteome. To identify infrequent and overrepresented words, we utilized the AW command line tool which estimates avoided and overabundant oligomers in biological sequences (58,59). Since all the significant absent peptides in our dataset are composed of five amino acids, we calculated the rarest as well as the most frequent pentamers in the human proteome (Table 5). Strikingly, significant human peptide MAWs are found at a high rate in non-eukaryotic organisms, whilst the most frequent sequences in the human proteome scarcely occur in bacteria, archaea, or viruses (Figure 4 and Supplementary Spreadsheet 6). Rarely occurring 5-mers follow a similar trend to MAWs, making this observation of importance for further investigation in the view of vaccine design. Ultimately, the results of this analysis support the conjecture that human MAWs might act as ‘fingerprints’ recognised by the immune system and corroborate the self-nonself discrimination paradigm (60–62).

### Functional consequences of human MAW-making mutations

**Figure 5.**
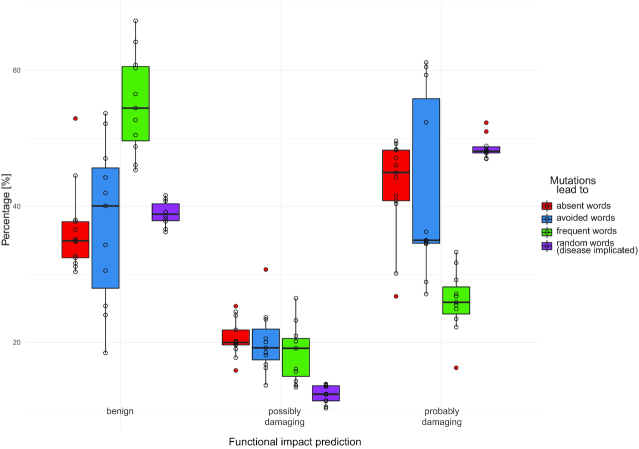
Prediction of mutational effects on protein function caused by amino acid replacements which generate (i) absent, (ii) avoided, (iii) frequent as well as (iv) disease implicated peptides in human proteins. Boxplots display the frequency of the predicted mutational effect (benign, possibly damaging, probably damaging) as predicted by the PolyPhen-2 algorithm. White circles depict the actual values of predictions while the outliers are shown as red circles. Each red box summarizes 13 datapoints (one per significant human MAW), each blue and green box summarizes 11 datapoints, and each purple box summarizes 10 datapoints.

To evaluate the impact of MAW-making amino acid replacements on the structure and function of the proteins, we exploited the ‘Batch query’ functionality of ‘Polymorphism Phenotyping v2’ (PolyPhen-2) using the default parameters as previously reported (63,64). We set out to investigate how human MAW-making mutations compare to amino acid substitutions which generate either rare or frequent motifs. We created two distinct lists of proteins which introduce any of the rarest/most common words in their sequences upon a single amino acid substitution. Additionally, we compiled 10 datasets of disease-implicated missense variants from the curated Humsavar catalogue of UniProtKB (https://www.uniprot.org/docs/humsavar) by randomly selecting 600 point mutations on average (∼8% of the total records in the dataset). We filtered out unreviewed protein records and kept only amino acid replacements that can occur upon a single nucleotide substitution in all the 4 datasets. Intriguingly, albeit disease-associated variants are confidently classified as ‘probably damaging’, we observe that MAW-making mutations have a proportionally less benign impact as compared to the other three categories (Figure 5). Mutations that lead to frequent motifs have a significantly less damaging effect in comparison with substitutions that generate rare words, indicating that suppressed sequences are not random and tend to be noxious. This observation signifies the importance of experimental validation on MAW-making sites, especially those that are predicted to have a detrimental effect. In order to further evaluate the harmfulness of MAW-making mutations, we retrieved functional impact annotation by Mutation Assessor, a server which predicts functional consequences of amino acid alterations (65,66). We compiled a list of 191 impactful MAW-generating mutations of reviewed proteins which are predicted to be highly damaging by both PolyPhen-2 and Mutation Assessor (Supplementary Spreadsheet 1). We retrieved data of human polymorphisms and somatic variants from COSMIC (67; https://cancer.sanger.ac.uk/cosmic), cBioPortal (68,69; https://www.cbioportal.org/), and Humsavar and asked whether any of the 191 amino acid alterations have known implications in diseases. Among the matching records (Supplementary Spreadsheet 1; see column ‘Involvement in disease’), the H886R mutation in Q12873 (*CHD3* gene) has been linked with the Snijders Blok-Campeau syndrome, while the H876R mutation in Q14839 (*CHD4* gene) has been correlated with prostate cancer. Interestingly enough, both mutations occur in the DEAH-box motif, while our list of probably damaging mutations include multiple recurrent H>R alterations at the same motif in other members of the DEAH-box protein family (see records O14646, O14647, Q86WJ1, Q8TDI0). We speculate that the ‘VDEAR’ MAW might be a pathological variant of the DEAH-box, a motif of RNA helicase proteins (70,71).

We further investigated whether MAWs occur in toxins or venom proteins, by exploiting the manually curated animal toxin annotation project (72; https://www.uniprot.org/program/Toxins). Three MAWs (‘AKITL’, ‘PTILA’, ‘TVIEL’) exist in five toxin records (UniProt IDs: O46028, P20798, A0RZC6, Q0ZZJ6, J3S836).

### Relative absent words and MAWs in viruses

**Figure 6.**
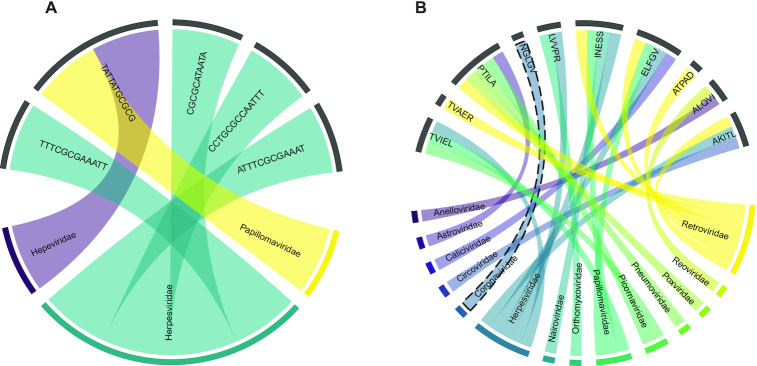
(**A**) Chord diagram presents five significant relative absent words, present in viral genomic sequences (virus families) but absent from the human genome. (**B**) Chord-diagram of correlations between human-derived peptide MAWs and virus families. The highlighted ‘*NGLGV’* MAW is not absent in sequences of *Coronaviridae*.

Recent studies have shown that viral sequences mimic those of their hosts, to some extent, in part to evade immune responses (30,73–74), and this can be used to predict viral hosts (31). To this end, we set out to survey virus genomes and proteins and investigate whether the scenario of RAWs fits with our previous human-derived findings. Viral sequences and annotation were downloaded from NCBI Virus (75) in March 2020. We filtered out incomplete sequences and records isolated from non-human species, resulting in 33 610 genomes and 233 178 proteins. Next, we queried whether significant human MAWs appear in any of the viral sequences. We observed that five genomic MAWs (out of the total 13) were present in 39 unique sequences of 3 distinct virus families (Figure 6A). What stands out is the remarkably low number of significant *human*-RAWs which are present in the virus genomes (Supplementary Spreadsheet 3). In other words, there is a general absence of absent human sequences that are present in viruses.

**Figure 7.**
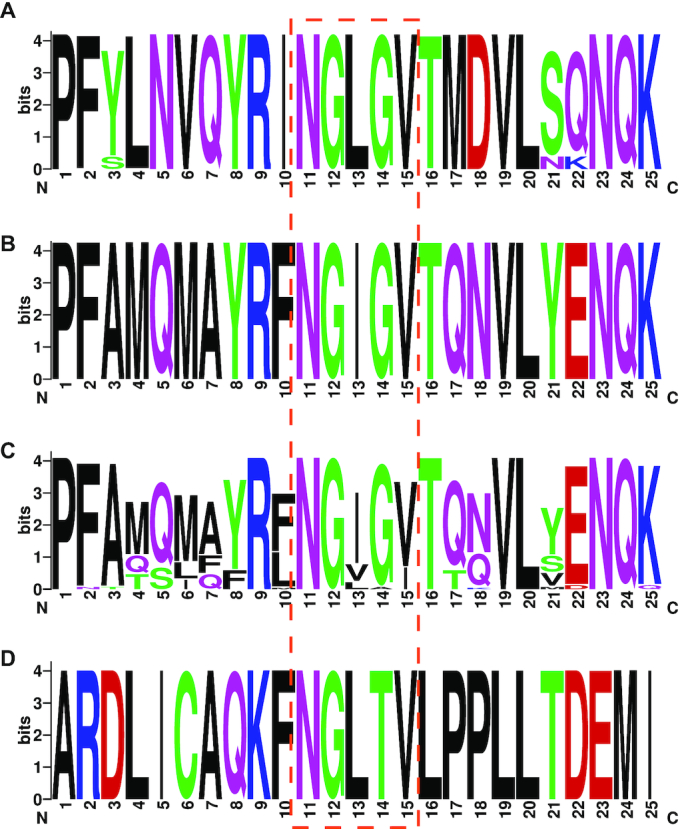
WebLogos of 25-amino-acid sequence windows from (**A**) 156 aligned spike glycoproteins of HCoV-HKU1 and HCoV-OC43 species in the region where the relative absent word ‘NGLGV’ occurs, (**B**) 435 aligned sequences from the spike glycoprotein of SARS-CoV-2 from the same protein region, (**C**) 71 aligned sequence windows from spike glycoproteins of various bat species and (**D**) five aligned sequences from spike glycoproteins of Betacoronaviruses extracted from pangolins.

Likewise, we investigated whether human-derived peptide MAWs are present in viral protein sequences. We observed that 10 out of the 13 significant human MAWs emerge in various proteins of 14 virus families (Figure 6B). Sequences of *Retroviridae* and *Herpesviridae* families share the highest number of RAWs with six and five motifs, respectively. Most interestingly, the relative absent peptide ‘NGLGV’ solely appears in 156 sequences of Human coronavirus HKU1 (HCoV-HKU1) and Human coronavirus OC43 (HCoV-OC43) which both belong to the *Betacoronaviruses* genus and tend to cause mild illness. After performing sequence alignment using Clustal Omega (76), we observed conservation of the ‘NGLGV’ motif in all 156 protein sequences of the family. More specifically, the ‘NGLGV’ pattern is localised in the S2 subunit of the coronavirus spike glycoprotein (77,78), a multifunctional protein which mediates virus invasion and fusion of the virion into host cells. We continued an *in silico* investigation on 435 full-sequenced spike glycoproteins of SARS-CoV-2 species from the current coronavirus pandemic (data retrieved on 9 April 2020 from NCBI Virus) and observed a similar (but not absent) motif ‘NGIGV’ within a similarly conserved sequence window of the same region. The replacement of human-absent ‘NGLGV’ by human-present ‘NGIGV’ suggests evolution towards host sequence mimicry and might contribute to immune evasion. Visual sequence weblogos (79) of 25-amino-acid windows around the specific RAWs (Figure 7A, B) demonstrate a clear amino acid consensus. Next, we extracted 71 records of full-sequenced spike glycoproteins of the same genus from various types of bat species (the latest collection date was March 2018). Although there is a varying pattern among these sequences, the most frequent motif in the examined region in bats is the ‘NGIGV’ again (Figure 7C) perhaps indicating evolutionary preadaptation. Moreover, only five sequences of spike glycoproteins from *Betacoronaviruses* in pangolins (*Manis javanica*) were available, which present a conserved ‘NGLTV’ oligopeptide within a dissimilar sequence window (Figure 7D). All protein sequences are provided in Supplementary Spreadsheet 3 (Supplementary Tables S3–S7). Although it remains unclear whether RAWs conceal underlying biological mechanisms, the fact that significant human-absent motifs appear in virus sequences may provide clues to reservoir species or viral toxicity.

Next, we examined whether viruses have unexpected missing motifs in their sequences. After filtering out non-complete sequences, we retrieved 147 799 individual virus genomes from NCBI Virus (data obtained in May 2020). We calculated minimal absent sequences for each virus separately and subsequently analysed them successively. More than 1 200 unique MAWs of length ranging from 4 to 10 residues were revealed significant upon Bonferroni correction at 1% cut-off. A wide species coverage is observed since the overall motifs are absent from >6000 distinct species whereas, interestingly enough, the most frequent missing sequences are recognition sites of host restriction enzymes, according to the Restriction Enzyme Database (80; http://rebase.neb.com/). Restriction enzymes present in prokaryotes recognise specific sequential patterns in viruses and cleave their DNA into fragments (81,82). These cutter enzymes provide a defence mechanism in host species against virus invasion and have been successfully utilized in a range of biotechnological applications and research studies (83–87). Although it is known that restriction sites confer a selective disadvantage to viruses, this has not been previously linked to nullomers or MAWs. The fact that our top hits include several palindrome sequences ranging from four to six bases in length offers potential for virus MAWs to be utilized as predictors of previously unknown restriction motifs in host organisms. Furthermore, given the robustness of our evaluation method, the entire dataset can offer a valuable resource for future *in silico* research. Table 4 presents the most frequent virus MAWs which are shared between hundreds of viral species while the entire list is provided in the form of a spreadsheet (Supplementary Spreadsheet 7) as well as through *Nullomers Database*.

**Table 4.** List of the most frequent (absent from >1000 distinct sequences) significant MAWs in viruses

MAW	Number of distinct sequences	Number of distinct species	Number of distinct genus	Number of distinct families	Virus family
**GCCGGC**	1580	1198	74	9	Ackermannviridae, Autographiviridae, Drexlerviridae, Marseilleviridae, Myoviridae, Pithoviridae, Podoviridae, Siphoviridae, Sphaerolipoviridae
**GGCGCC**	1536	1186	69	7	Ackermannviridae, Autographiviridae, Drexlerviridae, Myoviridae, Podoviridae, Siphoviridae, Sphaerolipoviridae
**GGGCCC**	1383	1084	58	8	Ackermannviridae, Autographiviridae, Marseilleviridae, Myoviridae, Pithoviridae, Podoviridae, Siphoviridae, Sphaerolipoviridae
**CCGCGG**	1379	1035	68	7	Ackermannviridae, Autographiviridae, Drexlerviridae, Myoviridae, Podoviridae, Siphoviridae, Sphaerolipoviridae
**CTGCAG**	1324	938	83	7	Ackermannviridae, Autographiviridae, Herelleviridae, Marseilleviridae, Myoviridae, Podoviridae, Siphoviridae
**GGATCC**	1242	953	72	9	Ackermannviridae, Autographiviridae, Demerecviridae, Herelleviridae, Marseilleviridae, Myoviridae, Pithoviridae, Podoviridae, Siphoviridae
**GATC**	1233	720	44	15	Adintoviridae, Autographiviridae, Corticoviridae, Herelleviridae, Inoviridae, Lavidaviridae, Lipothrixviridae, Microviridae, Myoviridae, Pleolipoviridae, Podoviridae, Siphoviridae, Sphaerolipoviridae, Spiraviridae, Tectiviridae
**CTCGAG**	1227	881	69	6	Autographiviridae, Demerecviridae, Herelleviridae, Myoviridae, Podoviridae, Siphoviridae
**CCCGGG**	1160	867	57	8	Ackermannviridae, Autographiviridae, Marseilleviridae, Myoviridae, Pithoviridae, Podoviridae, Siphoviridae, Sphaerolipoviridae
**GAGCTC**	1114	855	71	6	Ackermannviridae, Autographiviridae, Herelleviridae, Myoviridae, Podoviridae, Siphoviridae
**CAGCTG**	1058	788	45	6	Ackermannviridae, Autographiviridae, Herelleviridae, Myoviridae, Podoviridae, Siphoviridae

**Table 5.** List of the most (A) frequent 5-mers and (B) avoided 5-mers in the human proteome. The peptide search feature of UniProt (https://www.uniprot.org/peptidesearch/) was utilized to estimate the number of distinct proteins in which the overabundant/avoided motifs occur

**A**	Frequent sequences	Number of proteins contain the motif (Swiss-Prot)	Number of proteins contain the motif (TrEMBL)
	**CGKSF**	288	494
	**CGKTF**	250	397
	**CGKGF**	161	236
	**CGKAF**	411	673
	**HQRVH**	152	216
	**TGEKP**	525	965
	**YRDVM**	167	472
	**HERTH**	73	108
	**CGKVF**	134	198
	**HKRIH**	114	160
	**GEKPY**	481	915
			
**B**	Avoided sequences	Number of proteins contain the motif (Swiss-Prot)	Number of proteins contain the motif (TrEMBL)
	**LTGEK**	20	45
	**GEKPL**	21	61
	**GEKPS**	16	48
	**EGEKP**	4	9
	**TGEKG**	31	100
	**KGEKP**	15	36
	**GEKPK**	6	19
	**LGKAF**	11	18
	**GEKPT**	6	5
	**HTGEE**	9	25
	**PEKPY**	5	4

### Nullomers database

**Figure 8.**
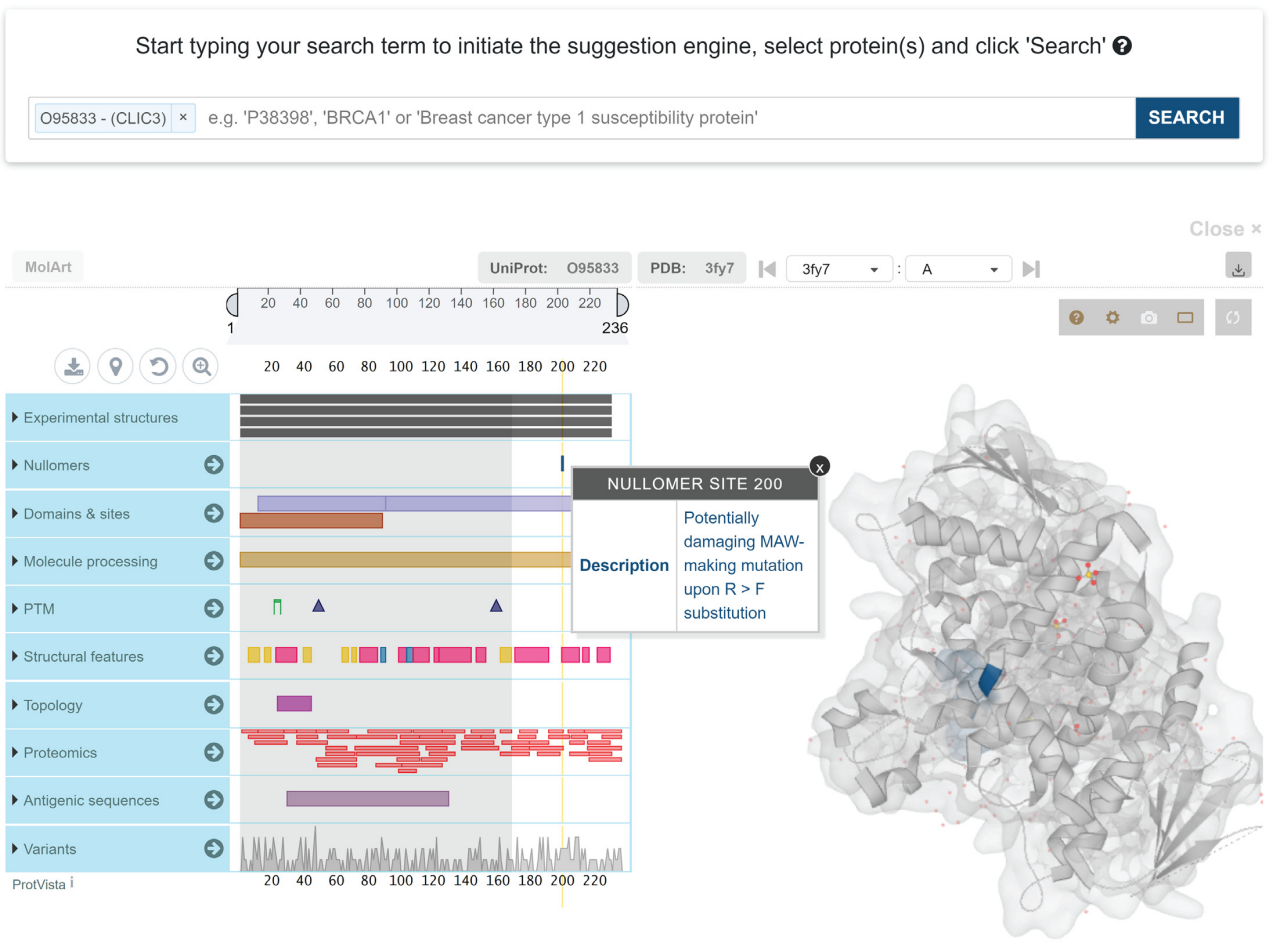
Snapshot from the graphical user interface (GUI) of *Nullomers Database*. Two interactive panels interconnect sequential annotation with tertiary structures offering a visual environment to explore MAW-making mutations in proteins of interest.

To make the MAWs of the current study easily accessible and explorable, we developed an online portal available at https://www.nullomers.org. *Nullomers Database* aims to serve as a central hub of information for further investigation of minimal absent words. The provided results are significant genomic and peptide MAWs assessed by *Nullomers Assessor*. Main emphasis has been given to peptide MAWs and, more specifically, to regions of proteins that are prone to give rise to a significant MAW upon a single amino acid alteration. In order to assess harmfulness of MAW-making substitutions, we provide functional impact annotation from Mutation Assessor. To prevent outdated information remaining in *Nullomers Database*, complex stored procedures have been developed in conjunction with an automated communication channel which retrieves information from UniProt. The gene name, protein sequence, description, sequential annotation as well as protein status (i.e. whether a protein is still active or has been deprecated and moved to UniProt Archive) are asynchronously collected from UniProt via a REST web-service. Given the dynamic nature of the UniProt database, the information retrieval of all the above-described steps has been automated, making *Nullomers Database* a fully autonomous, scalable, and frequently updated repository. Additionally, the integration of MolArt (88), an interactive visualization plugin (Figure 8), allows for the simultaneous exploration of multiple sequential and structural features in protein MAWs. The interconnected and synchronized panels of MolArt permit users to identify co-occurrent elements in regions that are prone to engender a missing word upon a single amino acid substitution. The entire sequence annotation of the plugin is retrieved from UniProt in real-time whereas the corresponding experimental structures are dynamically fetched from Protein DataBank (or from Swiss Model Repository when a structure is not available). Therefore, the specific functionality of *Nullomers Database* ensures that the provided information can always be in line with major protein databases and automatically be enriched over time.

## CONCLUSION

This paper introduces *Nullomers Assessor*, a probabilistic protocol provided as an open-source software tool, for the assessment of any set of minimal absent genomic or peptide sequences. The software offers a rigorous way of filtering missing words by Markov chains, while three statistical correction methods are available to control false positive results. We applied the script to entire genomes of hundreds of species and observed that numerous MAWs are statistically significant in multiple organisms. Moreover, we systematically examined >147 000 individual virus sequences and observed that the most frequent significant absent motifs are restriction recognition sites. In addition to the prevailing hypothesis that minimal absent words have gone extinct due to negative selection, we suggest that MAWs may have been replaced by more specialized sequences, which execute similar or even optimized functions.

We analysed the human and mouse proteomes and identified positions that are prone to introduce a significant missing peptide. We found that more than one-fourth of human proteins can give rise to a significant MAW upon a single amino acid substitution and showed that MAW-making mutations are often predicted to be damaging. We freely provide our findings in a visual, interactive, and user-friendly way via *Nullomers Database*. Taking advantage of the powerful functionalities that modern web technologies provide, we highlight protein positions which can generate a minimal absent word in their sequences.

In summary, the present study reveals significant MAWs that are unlikely to be absent by chance. Further studies should be conducted to experimentally validate and determine the actual role of MAWs as well as the extent of harmfulness behind MAW-making mutations; hence, we anticipate that both *Nullomers Assessor* and *Nullomers Database* can be useful resources and facilitate research towards a better understanding of the still mysterious role of minimal absent words.

## DATA AVAILABILITY

- **Project tools:** Nullomers Assessor / Nullomers Database
- **Project home page:** https://www.nullomers.org/
- **Source code:** https://github.com/gkoulouras/nullomers-assessor
- **Programming language(s):** Python, Microsoft ASP.NET, JavaScript
- **Operating system(s):** Platform-independent
- **Web browsers:** Google Chrome (v.87 or later), Mozilla Firefox (v.84 or later)
- **License:** Apache 2.0 (http://www.apache.org/licenses/LICENSE-2.0)

## Supplementary Material

gkab139_Supplemental_Files

## Notes

Present address: Grigorios Koulouras, Cancer Research UK Beatson Institute, Glasgow, UK and Institute of Cancer Sciences, University of Glasgow, Glasgow, UK.
