# Supplementary material for: Significant non-existence of sequences in genomes and proteomes
Source: Nucleic Acids Res. 2021 Mar 10;49(6):3139–55. doi: 10.1093/nar/gkab139 (PMC8034619; doi:10.1093/nar/gkab139)
Supplement: gkab139_Supplemental_Files [file gkab139_supplemental_files.zip › Supplementary_Material.docx]

**Significant non-existence of sequences in genomes and proteomes**

Grigorios Koulouras^1 #^ and Martin C. Frith^1, 2, 3 *^

^1^Artificial Intelligence Research Center, National Institute of Advanced Industrial Science and Technology (AIST), 2-3-26 Aomi, Koto-ku, Tokyo 135-0064, Japan.

^2^Graduate School of Frontier Sciences, University of Tokyo, Kashiwa, Chiba, Japan.

^3^Computational Bio Big-Data Open Innovation Laboratory (CBBD-OIL), AIST, Shinjuku-ku, Tokyo, Japan.

^#^ Present Address: Cancer Research UK Beatson Institute, Glasgow, UK and Institute of Cancer Sciences, University of Glasgow, Glasgow, UK

* Corresponding Author

Supplementary Material

| 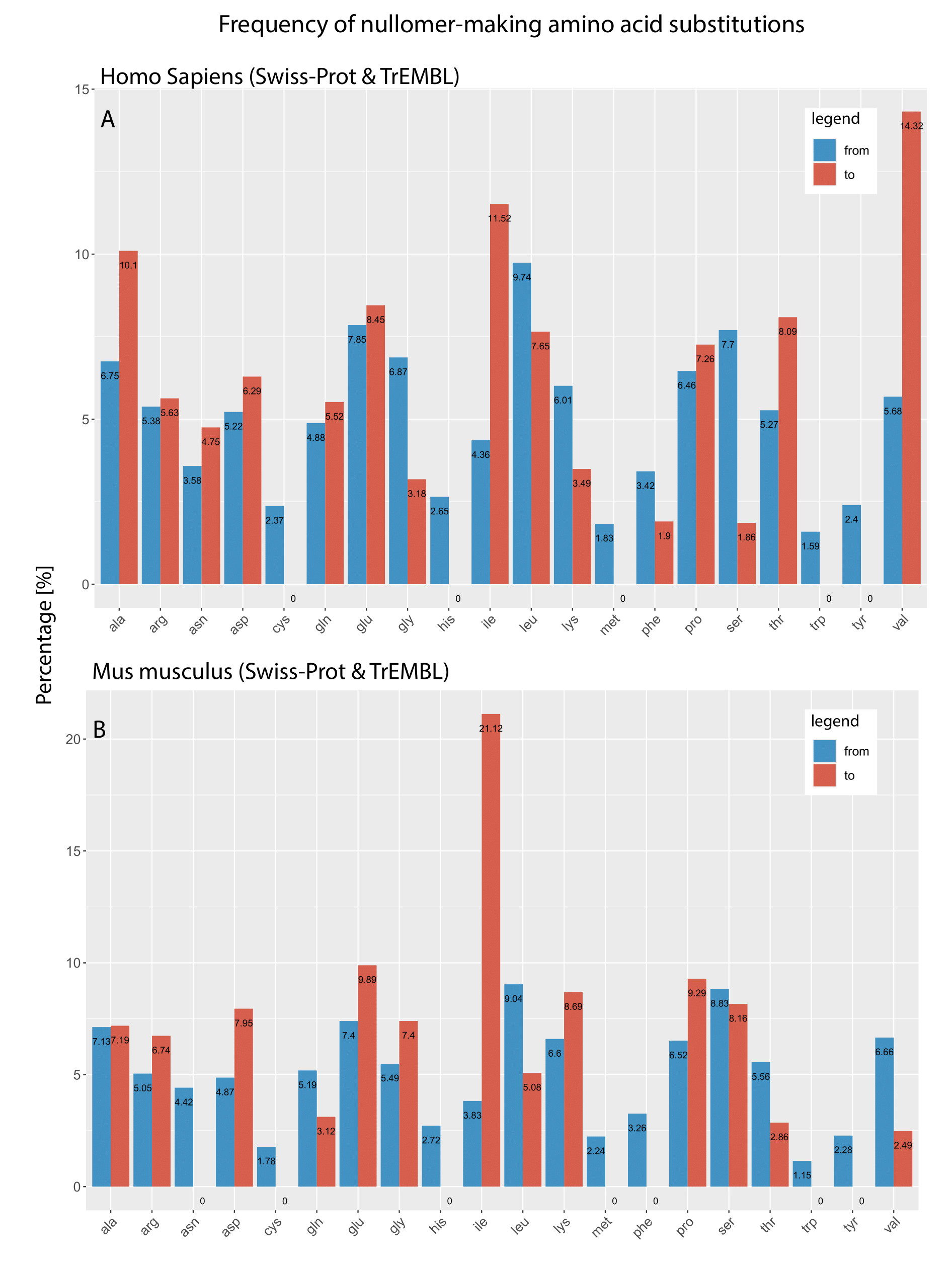 |
| --- |

**Supplementary Figure 1.** Proteome-wide analysis of all possible MAW-making mutations in (A) *Homo Sapiens* and (B) *Mus Musculus*. Each bar depicts the frequency of single amino acid alterations which generate a significant MAW. Blue bars represent the count of mutable amino acids that are prone to alter while red-coloured bars indicate the number of putative MAW-making substitutions. Proteomes include both canonical, isoform, reviewed and predicted records. Full proteome datasets retrieved from UniProt.

| 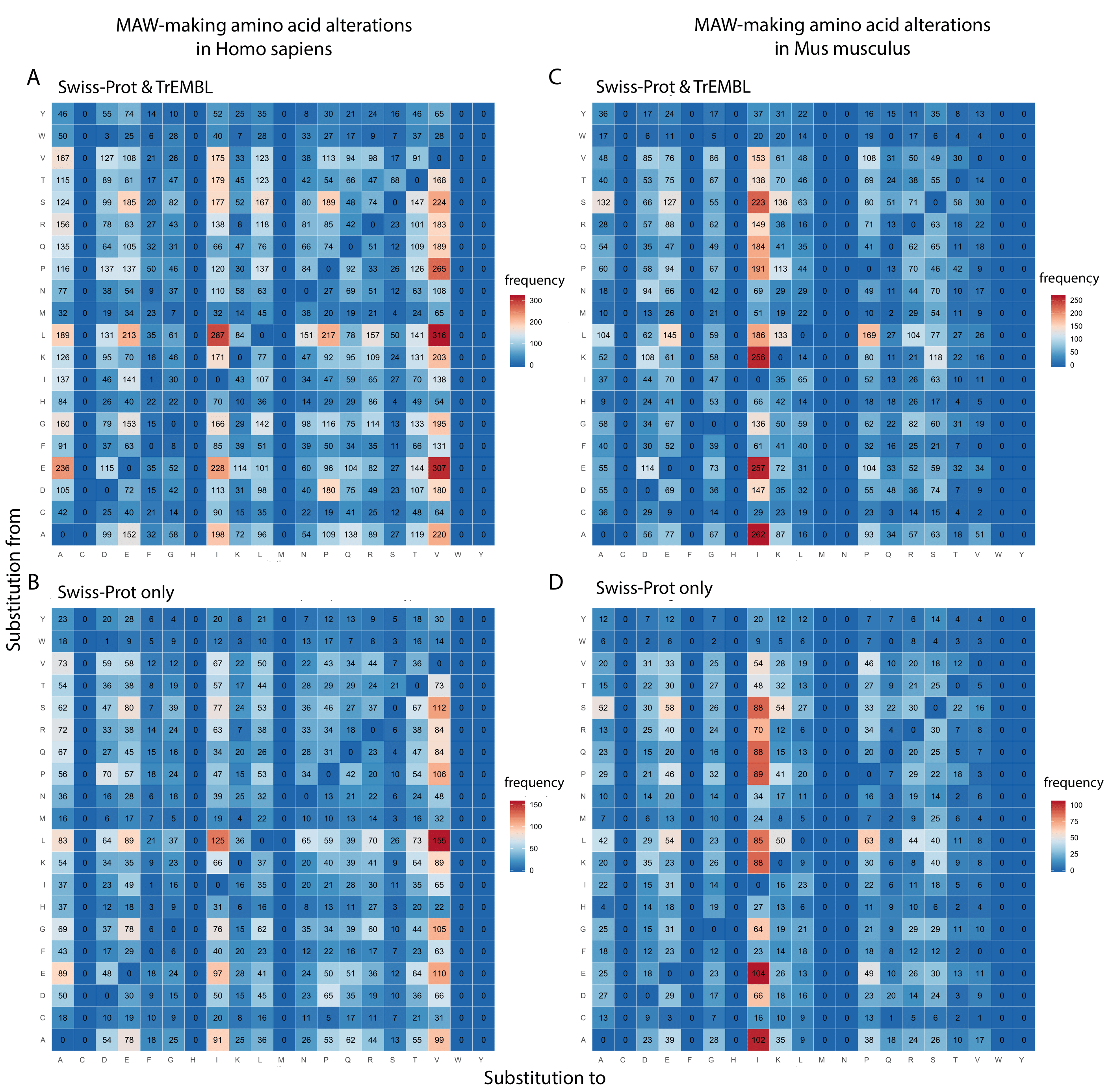 |
| --- |

**Supplementary Figure 2.** Proteome-wide mutational landscape of MAW-making alterations. Each grid-cell indicates the frequency of MAW-making alterations from one amino acid to another. The mutable amino acids are shown vertically, while the target amino acids are depicted in the horizontal axis. Subplot (A) presents MAW-making substitutions in human protein entries derived from Swiss-Prot and TrEMBL, while only curated records have been considered in (B). Similarly, (C) and (D) depict an identical analysis in Mus Musculus considering reviewed and predicted compared to curated-only records, respectively.

| 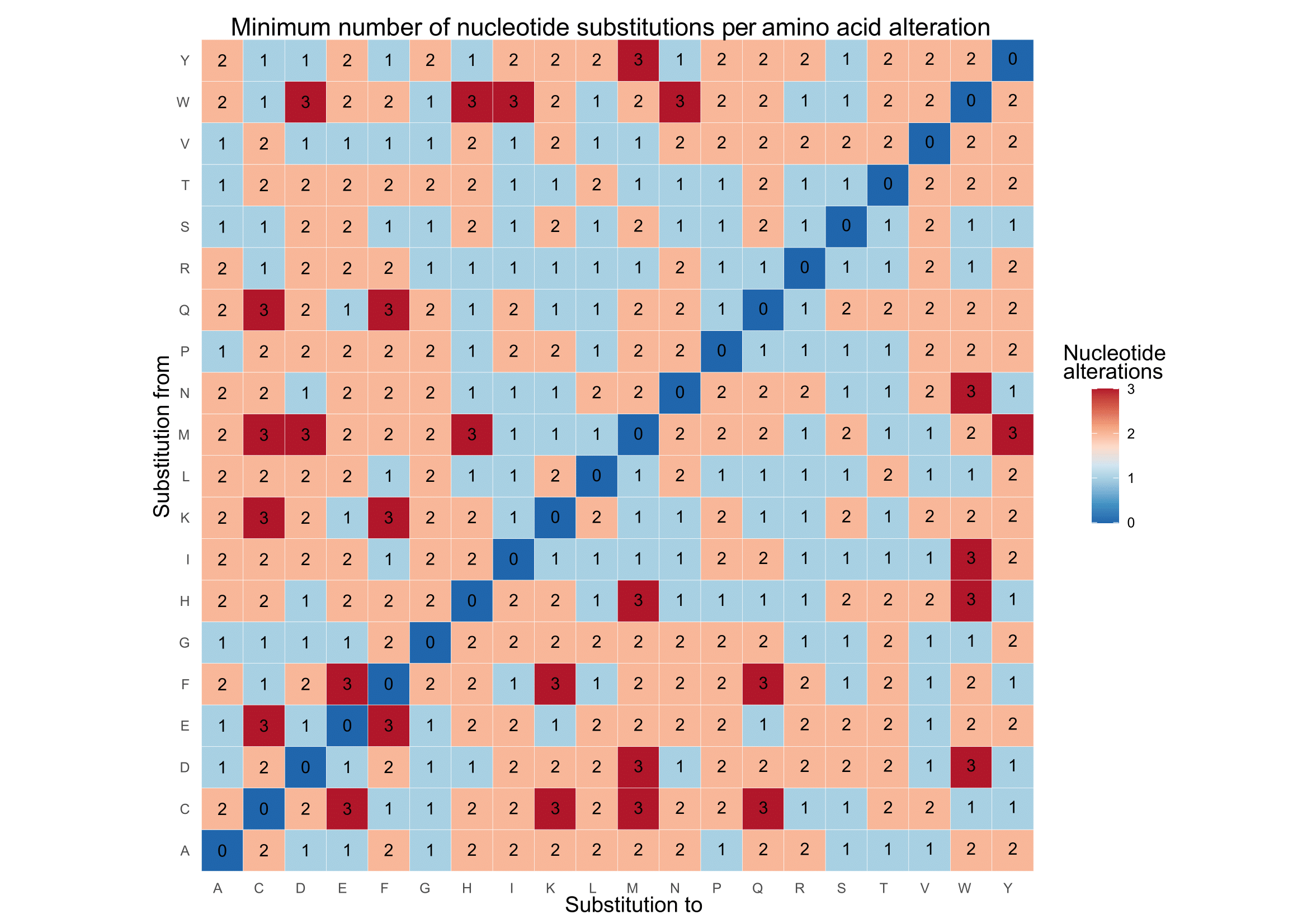 |
| --- |

**Supplementary Figure 3.** Square matrix shows the minimum number of nucleotide alterations that are required for amino acid replacements. MAW-making mutations that require a double-nucleotide or an entire codon change are the least probable to happen and possibly of lower importance.

| 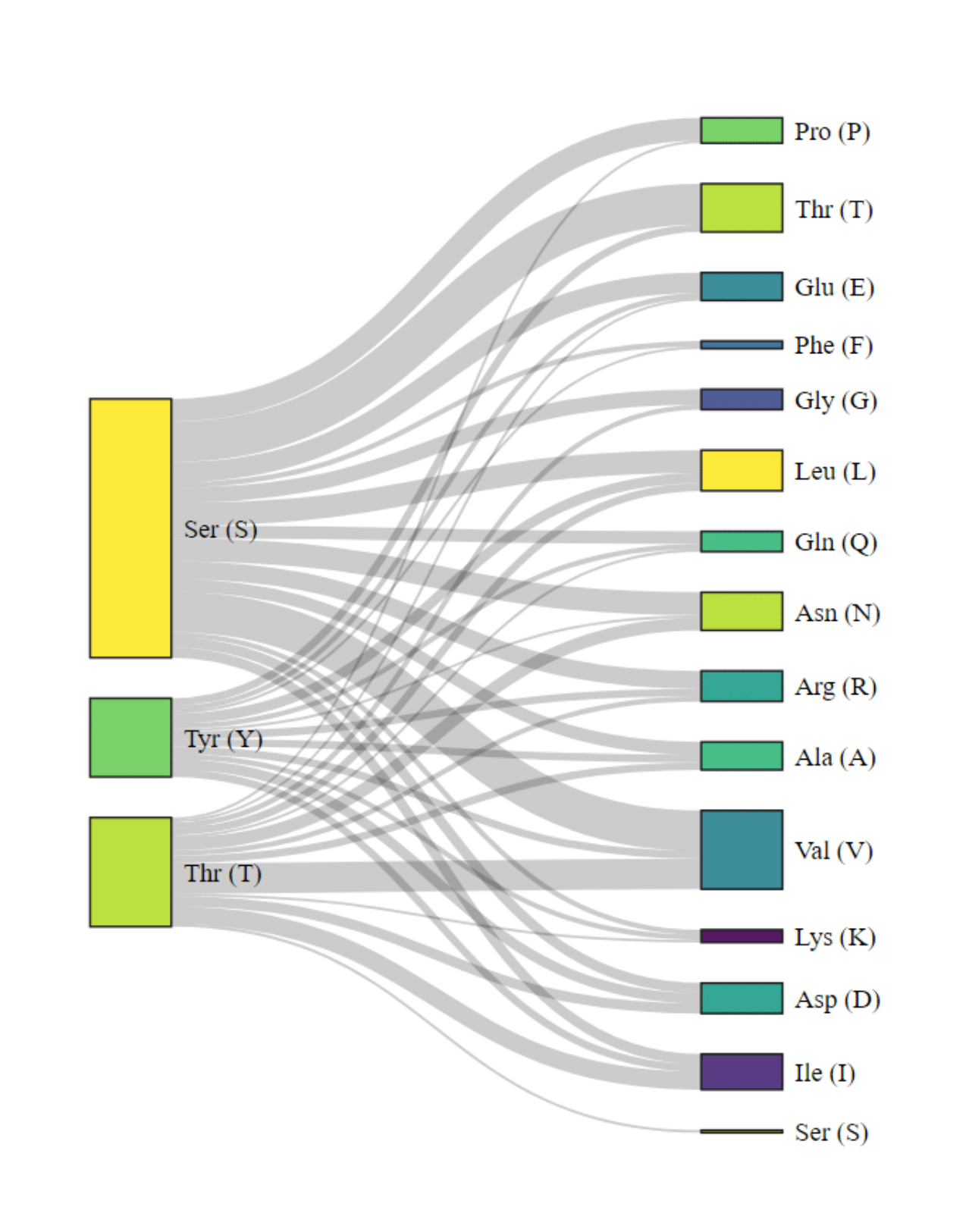 |
| --- |

**Supplementary Figure 4.** Sankey diagram illustrates MAW-making mutational trends in human phosphorylation sites. Experimentally verified protein phosphorylation sites have been extracted from PhosphoSitePlus® (v6.5.7) and subsequently matched with MAW-making mutations from Nullomers Database. The left-hand side residues are phosphosites susceptible to alter, while the targeted amino acids are presented in the right side. Box size proportionally indicates the number of putative alterations.

**Supplementary Table 1.** Summary table of poly-mononucleotide tracts* per length of significant genomic MAWs. Each table cell indicates the number of significant absent MAWs which contain a mononucleotide tract in their sequences.

| MAW length | Sequences contain a poly-A tract | Sequences contain a poly-C tract | Sequences contain a poly-G tract | Sequences contain a poly-T tract |
| --- | --- | --- | --- | --- |
| 9-mer | 79 | 10 | 10 | 93 |
| 10-mer | 46 | 4 | 3 | 63 |
| 11-mer | 18 | 1 | 0 | 19 |
| 12-mer | 0 | 0 | 0 | 1 |
| 13-mer | 0 | 0 | 0 | 0 |

* Since a poly-A tract always co-occurs with a same-length poly-T tract on the other strand, the poly-A and poly-T results can be considered complementary and interchangeable (likewise for C and G)
